# Compatible Mycorrhizal Types Contribute to a Better Design for Mixed *Eucalyptus* Plantations

**DOI:** 10.3389/fpls.2021.616726

**Published:** 2021-02-12

**Authors:** Fangcuo Qin, Shixiao Yu

**Affiliations:** State Key Laboratory of Biocontrol, Department of Ecology, School of Life Sciences, Sun Yat-sen University, Guangzhou, China

**Keywords:** plant-soil feedback, mycorrhizal type, species coexistence, *Eucalyptus* plantations, native tree species

## Abstract

Mixed-species forest plantation is a sound option to facilitate ecological restoration, plant diversity and ecosystem functions. Compatible species combinations are conducive to reconstruct plant communities that can persist at a low cost without further management and even develop into natural forest communities. However, our understanding of how the compatibility of mycorrhizal types mediates species coexistence is still limited, especially in a novel agroforestry system. Here, we assessed the effects of mycorrhizal association type on the survival and growth of native woody species in mixed-species *Eucalyptus* plantations. To uncover how mycorrhizal type regulates plant-soil feedbacks, we first conducted a pot experiments by treating distinct mycorrhizal plants with soil microbes from their own or other mycorrhizal types. We then compared the growth response of arbuscular mycorrhizal plants and ectomycorrhizal plants to different soil microbial compositions associated with *Eucalyptus* plants. We found that the type of mycorrhizal association had a significant impact on the survival and growth of native tree species in the *Eucalyptus* plantations. The strength and direction of the plant-soil feedbacks of focal tree species depended on mycorrhizal type. Non-mycorrhizal plants had consistent negative feedbacks with the highest survival in the *Eucalyptus* plantations, whereas nitrogen-fixing plants had consistent positive feedbacks and the lowest survival. Arbuscular mycorrhizal and ectomycorrhizal plants performed varied feedback responses to soil microbes from distinct mycorrhizal plant species. Non-mycorrhizal plants grew better with *Eucalyptus* soil microbes while nitrogen-fixing plants grew worse with their own conspecific soil microbes. Different soil microbial compositions of *Eucalyptus* consistently increased the aboveground growth of arbuscular mycorrhizal plants, but the non-mycorrhizal microbial composition of the *Eucalyptus* soil resulted in greater belowground growth of ectomycorrhizal plants. Overall, *Eucalyptus* plants induced an unfavorable soil community, impeding coexistence with other mycorrhizal plants. Our study provides consistent observational and experimental evidence that mycorrhizal-mediated plant-microbial feedback on species coexistence among woody species. These findings are with important implications to optimize the species combinations for better design of mixed forest plantations.

## Introduction

Increasing demand for timber products has led to the worldwide expansion of plantations of fast-growing tree species. However, these plantations are usually established in monoculture and are dominated by a few genera, such as *Eucalyptus*, *Pinus*, and *Acacia*, in tropical and subtropical regions. This has led to a series of ecological problems, such as soil degradation and erosion, loss of local plant diversity, and decrease in ecosystem functions (Bauhus et al., 2017). To resolve the dilemma, screening suitable plants is vital for a better design of mixed forest plantations that increase plant diversity and restore original ecosystem function and the degraded pure plantations (Forrester et al., 2006; Amazonas et al., 2018; Yao et al., 2019). However, the compatible combinations of intercropped species and the mechanism underlying species coexistence among different woody plants in afforestation ecosystems remain poorly understood.

Plant-soil feedback (PSF) plays an important role in terrestrial plant coexistence, diversity and community succession (Mangan et al., 2010; van der Putten et al., 2016; Pizano et al., 2019). PSF refers to plant-induced species-specific changes in soil biotic and abiotic conditions, which in turn impact the growth and recruitment of subsequent plants (van der Putten et al., 2013). Focal species may be negatively affected by reduced nutrient availability and the accumulation of pathogens and allelochemicals, whereas they may be positively affected by the promotion of symbionts and/or nutrient availability (Bennett and Klironomos, 2019; Wang et al., 2019). The relative strength and direction of PSFs can depend on the results of positive vs. negative feedbacks, which have implications for the plant community assembly (van der Putten et al., 2013; Liang et al., 2015; Chen et al., 2019).

Mycorrhizal associations with plant roots are important drivers of positive PSF (van der Putten et al., 2013). Currently, the positive feedback mediated by mycorrhizal fungi tends to result in local dominance through promotion of their host performance (Peh et al., 2011). Mycorrhizal fungi provide host plants with soil nutrients and antagonists protection in exchange for photosynthates. There is also a host preference in mycorrhizal symbiosis ranging from facultative to obligate (Smith and Smith, 2015), due to preferential allocation of photosynthates to more efficient mycorrhizal fungi partner by host plants (Bever, 2015). Moreover, the benefits derived from mycorrhiza may be contingent upon mycorrhizal type and may lead to species-specific PSF promoting species coexistence (Bennett et al., 2017).

Mycorrhizal type, through impact on soil microbial community, alter the interspecific competition and facilitation effects of co-existing plants. Mycorrhizal type that improves plant coexistence is usually linked to the complementary use of soil nutrient resources (Turner, 2008; Luo et al., 2018), such as nitrogen (N) and phosphorus (P). Mycorrhizal type of dominant tree species is a potential predictor of the biochemical process of soil nutrient dynamics by which selecting for specific microbial groups with distinct enzyme functions (Cheeke et al., 2017). This will induce a negative PSF on plant growth and provide the dominant plants a competitive advantage over neighboring or subsequent plants. When limited to the same resources, the functional diversity of mycorrhizal types could relieve competitive pressure and enhance plant growth and survival. A recent study has showed that partitioning of soil P among different mycorrhizal trees contributed to species coexistence in subtropical natural forests (Liu et al., 2018). In addition, a growing body of literature indicates that the composition of aboveground plant communities can culture the belowground microbial community toward certain functional groups, such as species-specific pathogens (Wardle et al., 2004; Bever et al., 2010; Yang et al., 2020). Plant functional traits have also been suggested to affect adaptation to the local soil chemistry or soil microbial community (Revillini et al., 2016). For example, ectomycorrhizal (EM) plants may represent more positive PSFs than arbuscular mycorrhizal (AM) plants because EM plants have greater access to transfer N *via* the ectomycorrhizal networks (Simard et al., 2012; Tedersoo et al., 2020) or greater protection from antagonists through the physical sheath that surrounds young feeder roots (Bennett et al., 2017). The bacteria community in the mycorrhizosphere also has significant impact on spore germination or colonization of mycorrhizal fungi (Xavier and Germida, 2003). Thus, the soil microbial community cultured by different mycorrhizal type of focal plants may lead to species-specific feedbacks, resulting in species coexistence or exclusion with subsequent plants.

*Eucalyptus*, indigenous to Australia and Indonesia, is the most common afforestation or reforestation genus in the world (Forrester, 2013). China has the second largest area of *Eucalyptus* plantations, which covered 4.5 million hectares by the end of 2014 (Chinese Society of Forestry, 2016). However, *Eucalyptus* plantations strongly alter the native plant community structure and function (Gaertner et al., 2011) and produce unfavorable soil conditions for subsequent plant species (Tererai et al., 2013; Wu et al., 2013). Many studies have suggested that mixing fast-growing leguminous tree species (e.g., *Acacia*) with *Eucalyptus* is a good silvicultural practice that increases plant diversity and soil nutrients (Forrester et al., 2006; Santos et al., 2017; Voigtlaender et al., 2019). However, we found that legume-rhizobium symbiosis is susceptible to allelopathic inhibition caused by *Eucalyptus* allelochemicals (Liu et al., 2019), resulting in worse survival of leguminous plants beneath *Eucalyptus* plantations than other mycorrhizal tree species (Qin et al., 2018). Moreover, exotic fast-growing species is also a threat of biological invasion. Thus, it is vital to screen compatible species that can coexist with *Eucalyptus* for a better design of mixed *Eucalyptus* plantations (Tesfaye et al., 2015; Sun et al., 2017).

Recent evidence has suggested that the mycorrhizal type of host plants can determine the strength and direction of the plant-soil feedback in Mediterranean shrublands and temperate forests (Bennett et al., 2017; Teste et al., 2017). However, the relative benefit of different mycorrhizal types can be context-dependent. *Eucalyptus* plants are capable of associating with both AM fungi and EM fungi (Chen et al., 2000; Teste et al., 2019), yet are powerful model plant systems to better understand the role of mycorrhizal symbioses in forest restoration and degraded plantation reconstruction. In the case studies of natural forests, the dominant mycorrhizal type has substantial impacts on subsequent or neighboring plants, by selecting for microbial groups with different nutrient cycling processes and protection from antagonists (Cheeke et al., 2017; Jo et al., 2017). Little is known about the contrasting effects of distinct mycorrhizal type on species coexistence in an afforestation system. Here, we test the importance of mycorrhizal type in plant-soil feedback and its influence on species coexistence in mixed *Eucalyptus* plantations. We hypothesized that the compatibility of different mycorrhizal types, through effects on the adaptation to soil microbial community, would account for interspecific competition exclusion or coexistence. For this, we first conducted a 10-year field survey in a mixed *Eucalyptus* plantation to estimate the effects of mycorrhizal type on the survival and growth of 20 woody species. We then conducted pot experiments to determine how mycorrhizal type determines PSF, by inoculating different types of mycorrhizal tree species with their conspecific or heterospecific soil microbes. Furthermore, we tested the contrasting effects of different compositions of soil microbes induced by *Eucalyptus* plants on AM and EM plants. We hypothesized that, (i) mycorrhizal type of tree species significantly influences their survival and growth in *Eucalyptus* plantations, (ii) plant-soil microbial feedback mediates the species coexistence between *Eucalyptus* and other mycorrhizal tree species, and the feedback strength depends on mycorrhizal type, and (iii) *Eucalyptus* is more likely to coexist with plants of more different mycorrhizal type.

## Materials and Methods

### Study Site

Our field site is located in Shuilian Mountain Forest Park, Dongguan city, Guangdong Province, China (113°42′E, 22°58′N). The region has a subtropical monsoon climate. The mean annual precipitation is 1,780 mm, and the rainy season extends from April to September. The mean annual temperature is 23.2°C. Soils are latosols developed on granite with a pH of 3.8. The original vegetation was subtropical monsoon evergreen broad-leaved forest, including the families of Euphorbiaceae, Lauraceae, and Theaceae. However, the original vegetation had almost disappeared in 1990s as a result of the long-term human disturbance and severe soil erosion. The site was cleared of all vegetation (mainly shrubs and herbs) prior to reforestation of *Eucalyptus urophylla* plantations in 1992. The *E. urophylla* plantations covered about 200-ha and served as ecological welfare forests, and subsequently protected from any human disturbance after establishment. By the end of 2006, *E. urophylla* trees entirely dominated the canopy and many dead adult *E. urophylla* individuals were found in the plantations. Only a few native tree species were distributed sporadically in the plantations, such as *Diospyros morrisiana*, *Rhus sylvestris*, and *Aporosa chinensis*. The understory was occupied by shrub species (e.g., *Psychotria rubra*, *Ilex asprella*) and herbaceous species (e.g., *Scleria levia*, *Microstegium vagans*). The pot experiments were conducted in the greenhouse at the School of Life Sciences building, Sun Yat-sen University, Guangzhou Higher Education Mega Center, Guangzhou city, with similar climate conditions to Shuilian Mountain Forest Park. The green-house had the sunlight and was at a day/night rhythm of temperatures of 28/22 °C in summer, and the air humidity was 75%.

### Field Survey

To estimate the effects of mycorrhizal association type on survival and growth of tree species in *Eucalyptus* plantations, we established a permanent plot at the field site during winter 2006 to spring 2007. Initially, we collected seeds as many as 56 broad-leaved tree species. Those seeds were placed in plastic basins with buck soil for germination and grew for 6–8 weeks before transplantation. However, based on the quantity and quality of seedlings, we chose 20 broad-leaved tree species and transplanted them into the *E. urophylla* plantations. According to published data (Wang and Qiu, 2006; Brundrett, 2009; Akmetzhanova et al., 2012) and results of our observations on their fine roots, we classified these 20 species into four root mycorrhizal types, namely, (i) AM, (ii) EM, (iii) nitrogen-fixing (NF), and (iv) non-mycorrhizal (NM) plants (Supplementary Table 1). We cleared all understory vegetations in the *E. urophylla* plantations and set up six parallel transects (15 m × 95 m for each) along the contours of the hillside. Each transect included six plots (10 m × 10 m) with 5 m-wide buffer zones between each plot, and 4–6 adult *E. urophylla* trees were left in every plot. We selected 10 tree species and transplanted them into a plot, at a density of 1 seedling/m^2^ (i.e., 100 seedlings per plot). In each transect, every three out of six plots was planted with the same 10 species while the other 10 species were planted into the rest of three plots (Supplementary Figure 1). Thus, there were 3,600 seedlings in total in this planting experiment (i.e., 6 transects × 6 plots/transect × 100 seedlings/plot). We replaced wilted or dead seedlings during the first 30 days after planting. We tagged all seedlings and first measured the basal diameter of each seedling in February 2007. Thereafter, we recorded the survival status of each seedling for each tree species and remeasured their basal diameter every 3 months. The last survey was conducted in June 2016.

### PSF Experiments

We conducted two pot experiments using germinated seedlings to uncover the impact of mycorrhizal type on PSFs between *Eucalyptus* and broad-leaved tree species. We collected seeds of the focal native tree species during autumn and winter in 2016 from Heishiding Nature Reserve and Dinghushan Nature Reserve, Guangdong Province, China. *A. lebbeck* is an introduced NF species that is used to improve soil fertility (Forrester et al., 2006; Hoogmoed et al., 2014). Seeds of target species were surface sterilized (1 min 70% ethanol, 3 min 2.625% NaClO, 1 min 70% ethanol, and 1 min distilled water). We dried the seed surface at room temperature and stored them at 4°C until March 2017. We sowed seeds into plastic boxes for germination in wet sterilized buck soils in the greenhouse. Newly germinated seedlings (2–4 weeks) were chosen for transplantation as the experimental treatments. *E. urophylla* is one of the most common afforestation tree species in southern China, and we obtained *E. urophylla* seedlings from the China Eucalypt Research Centre, Zhanjiang city, Guangdong Province. Before we transplanted the seedlings into the pots, we measured the fresh weight of each seedling. In addition, for each species, we randomly selected 30–40 seedlings and measured their fresh weight and dry weight (65°C for 48 h). We established the allometric growth relationships between fresh weight and dry weight to determine the initial dry weight of each planted seedling.

PSF Experiment 1: To illustrate how mycorrhizal type associated with the specific soil microbial community determines PSF strengths, we conducted a pot experiment using germinated seedlings. We chose nine co-existing tree species in the first planting experiment as focal species, namely 3 AM plants (*Cinnamomum camphora*, *Pterospermum lanceaefolium*, and *Schima superba*), two EM plants (*Castanopsis fissa* and *Castanopsis chinensis*), two NF plants (*Albizia lebbeck* and *Ormosia glaberrima*), one NM plant (*Helicia cochinchinensis*), and one eucalypt (EU) plant (*E. urophylla*) (Supplementary Table 1). For each focal species, we selected six adult trees and collected two soil samples from the root zones of each individual (<0.5 m from the base of the stem, 0–20 cm depth), and thoroughly pooled all samples for its species-specific soil. To avoid cross-contamination, we cleaned the shovels and sterilized them with 70% ethanol prior to the next sampling. In addition, we collected bulk soil from the field site, where those target tree species was not present within a radius of 50 m. To prepare the background soil, we mixed the bulk soil with sand (v 1:1) followed by sterilization with gamma radiation (25 kGy). We then thoroughly mixed the background soil with the species-specific soil (v 9:1) to prepare seven soil inocula treatment for each species: (i) conspecific sterilized soil, (ii) conspecific living soil, (iii) mixed soil of all species with the same mycorrhizal type to focus species and (iv)-(vii) the other four types of soil, respectively, mixed with their species-specific inocula (Supplementary Figure 2A). In particular, the NM and EU type with only one species, so *H. cochinchinensis* and *E. urophylla* seedlings both had only six inocula treatments without the mixed inocula of (iii). For each species, we filled plastic pots (13 cm in diameter, 15 cm in height) with one of 6 or 7 soil inocula in each pot. Thus, seedlings of the nine focal species were treated with 61 soil inocula treatment combinations and there were 549 pots in total (Supplementary Figure 2A), namely (7 species × 7 soil inocula + 2 species × 6 soil inocula) × 9 replicates.

PSF Experiment 2: To evaluate the effects of different microbial compositions in soil cultured by *Eucalyptus*, we used four AM plants (*Canarium album*, *C. camphora*, *Cryptocarya concinna*, and *Hovenia dulcis*) and four EM plants (*Castanopsis faberi*, *Castanopsis fissa*, *Cyclobalanopsis bambusaefolia*, and *Cyclobalanopsis fleuryi*) for focal plants. We separately collected species-specific *Eucalyptus* soil from three common *Eucalyptus* species (i.e., *E. urophylla*, *Eucalyptus dunnii*, and *E. urophylla* × *Eucalyptus grandis*), at eight adult individuals per species. To separate different compositions of the soil microbial community of *Eucalyptus*, we mixed those *Eucalyptus* soils and successively passed them through 250, 45, and 20-μm sieves, using the wet-sieving method (Klironomos, 2002). The first soil suspension that passed through the 250-μm sieve was treated as field soil (i), representing the composition and abundance of the whole soil microbial communities in *Eucalyptus* soil; and then, we extracted the spores of arbuscular mycorrhizal fungi (mainly) for mycorrhizal soil fractions of mycorrhiza (ii), from the leftover remained on the 45-μm sieve; the final suspension that passed through the 20-μm sieve was used for non-mycorrhizal soil fractions of without mycorrhiza (iii). We sterilized the suspension passed through 250-μm sieves by gamma radiation (25 kGy) and treated it as a control for abiotic effects (vi). We inoculated the newly germinated seedlings with those four microbial inocula (Supplementary Figure 2B). Thus, this experiment contained 512 pots in total, namely (8 species × 4 soil inocula × 16 replicates).

In the two PSF experiments, we first filled the pots with soil (i.e., sterilized background soil + soil inocula) until ∼2 cm from the top of the pots. We then transplanted one seedling into a pot and added extra sterilized background soil (1 cm) to each pot to prevent air contamination. One week after seedling transplantation, we removed the seedlings that were dead or growing poorly and replaced them with new conspecific seedlings. We then measured the initial height and number of leaves of each seedling. Plants were watered twice a week using tap water and allowed to grow for 8 months. We randomly arranged the treatments within each block and exchanged the positions of each pot every month to decrease environmental heterogeneity. After measuring the final height and number of leaves of each living seedling, we harvested the plants and determined the dry weight of shoots and roots for every seedling. Three pieces of intact and mature leaves from each seedling were chosen and used to determine the specific leaf area (SLA) (Cornelissen et al., 2003).

### Soil Fungal Community and Soil Nutrient Characteristics

To determine difference on soil microbes and soil nutrients of the nine focal tree species in *Experiment 1*, we collected three species-specific soil samples for each focal species. Every soil sample was divided into two parts. One part of 5-g soil was used for analyzed the soil fungal community. The total genomic DNA was extracted from each sample by a standard protocol using CTAB (cetyl trimethylammonium bromide) (Allen et al., 2006). The nuclear ribosomal internal transcribed spacer region (ITS rDNA gene) was amplified by polymerase chain reaction (PCR) using the fungal primer set ITS1-1F (5′-CTTGGTCATTTAGAGGAAGTAA-3′) and ITS2 (5′-GCTGCGTTCTTCATCGATGC-3′). PCR products were mixed in equal density ratios and then purified with a Qiagen Gel Extraction Kit (Qiagen, Germany). DNA samples were sequenced on an Illumina HiSeq2500 platform (Illumina, San Diego, CA, United States). The sequencing libraries were generated using a TruSeq DNA PCR-Free Sample Preparation Kit (Illumina, United States). Raw tags data were demultiplexed, quality-filtered and merged by FLASH (V1.2.7^1^) (Magoć and Salzberg, 2011). Standard quality control procedures for tags data were conducted by the BLAST taxonomy assignment method in QIIME (V1.7.0^2^) (Caporaso et al., 2010). We identified fungal operational taxonomic units (OTUs) with 97% similarity cutoff using UPARSE (version 7. 01^3^) (Edgar, 2013) and removed chimeric sequences using UCHIME (Edgar et al., 2011). The taxonomy of each sequence was analyzed by the RDP Classifier algorithm (version 2.2^4^) against GreenGene Database^5^ (DeSantis et al., 2006).

The other part of soil sample was air-dried at room temperature (25°C) and sieved through a 2-mm mesh. We then measured pH and the content of soil organic carbon (SOC), total nitrogen (TN), total phosphorus (TP), total potassium (TK), available nitrogen (AN), available phosphorus (AP), and available potassium (AK).

### Data Analysis

For the *Field survey*, we conducted analysis of variance (ANOVA) to detect the effects of mycorrhizal type on plant survival and basal diameter growth of the four types tree species at 18, 36, 54, and 114 months since planting; we then compare their average survival rates between those four mycorrhizal types during the four planting periods, respectively. We calculated the survival for each species in each plot. The basal growth of each living individual was calculated as follows: (logDt − logD0)/t, where D0 was the initial basal diameter and Dt was the basal diameter measured at time t after planting.

For the *PSF Experiment 1*, we detected how mycorrhizal type determines the strength and direction of PSFs. PSF was calculated by comparing relative growth of seedling that in its conspecific soil inocula (Con) relative to that growth in heterospecific soil inocula (Heter) according to the mycorrhizal types: PSF = log (Con/Heter). A negative feedback indicates the promotion of coexistence while positive feedback indicates monodominance. The relative growth of each seedling by the change in dry weight, which was calculated as the final dry weight of surviving seedlings divided by its estimated initial dry weight. The gain in dry weight for every seedling of each individual species was calculated as the harvested dry weight minus the estimated initial dry weight. We conducted two-way ANOVA to determine the effects of mycorrhizal type (M), inoculum source (I) and their interaction (M × I) on the gain in dry weight (gainDW), height (gainH), number of leaves (gainNL), and SLA of target species. For the soil fungal community in the species-specific soil of nine focal species, we constructed a Venn diagram to show shared or unique microbial species among them, using the Venn Diagram package in R 3.3.3.

For the *PSF Experiment 2*, we proposed the microbial growth response (MGR) to estimate effects of different microbial compositions in *Eucalyptus* soil on seedling height, total biomass and ratio of above-/below-ground dry weight (AbUn). The MGR referred to the net effect of soil biota (i.e., growth with versus without soil microbes) and was calculated using the following formula: MGR = log (growth in live soil/growth in sterilized soil). Negative and positive MGR values, respectively, indicate the negative and positive effects of soil microbes on seedling growth.

We log10-transformed the PSFs and MGRs. To fits statistical assumptions (i.e., normality and homogeneity of variance for the data set), we conducted bootstrapping (*n* = 1,000) for the resulting mean and variance statistics of PSF and MGR. It was considered to be significantly positive or negative when their 95% confidence intervals (CIs) for the mean values did not overlap zero. Tukey’s HSD (*P* < 0.05) was used for multiple comparisons. Statistical procedures were conducted in R for Windows 3.3.3 (R Development Core Team, 2016).

## Results

### Field Survey: Survival and Growth of Different Mycorrhizal Tree in *Eucalyptus* Plantations

**FIGURE 1:**
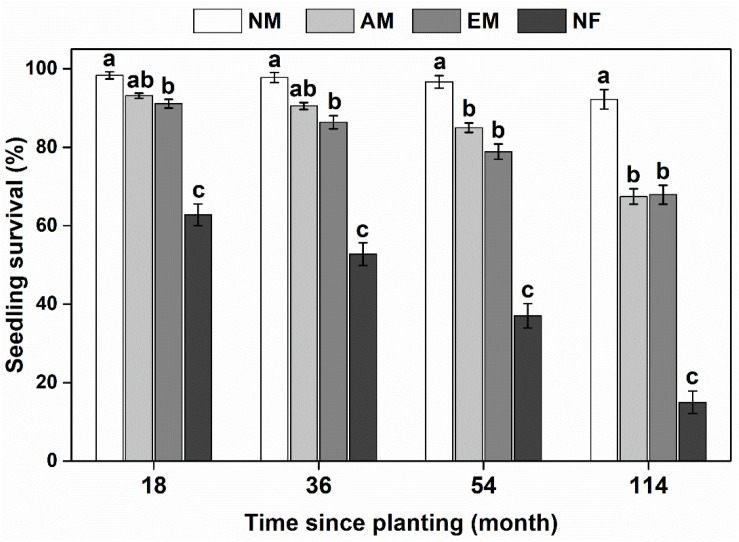
Survival rate of woody species with different mycorrhizal types in the *E. urophylla* plantations. NM, non-mycorrhizal; AM, arbuscular mycorrhizal; EM, ectomycorrhizal; NF, nitrogen-fixing. Values are the means ± SE. Bars with different letters are significantly different based on Tukey’s HSD tests (*P* < 0.05).

In the field, mycorrhizal type had a significant impact on survival and basal growth of focal tree species, either in the short term (18 months) or relative long term (114 months) (Figure 1 and Supplementary Table 2). Mycorrhizal plants survived better than NF plants. NF plants (e.g., *L. leucocephala* and *A. lebbeck*) suffered the lowest survival rate with only an average of 15% after 10 a since planting (Supplementary Figure 3). There was no significant difference between AM and EM tree species on seedling survival. NM species (i.e., *H. cochinchinensis*) consistently had the highest survival rate compared with mycorrhizal and NF species (Figure 1).

### PSF Experiment 1: Effects of Mycorrhizal Type on PSFs

**FIGURE 2:**
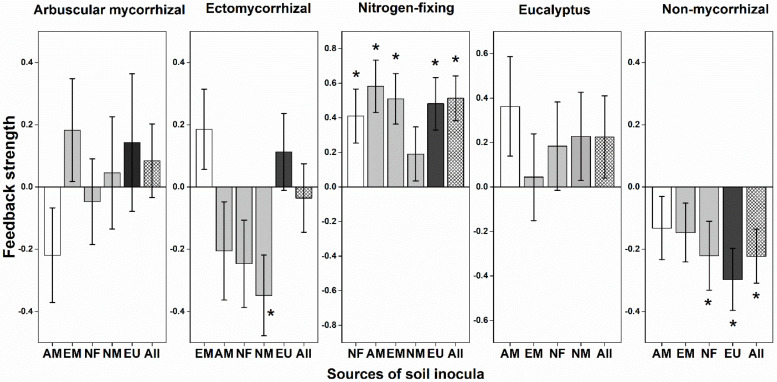
Plant-soil feedback (PSF) strength in relation to mycorrhizal types. Feedback strength is shown as log10 response ratios of the variation of plant biomass in conspecific (Con) with heterospecific (Heter) soils: PSFs = log10 (Con / Heter). Variation of plant biomass was calculated as the dry weight after harvested at the end of the experiment divided by the dry weight of the same seedling according to the allometric growth equation. The positive PSFs suggest species coexistence, while the negative PSFs suggest monodominance. Bootstrapping procedures (*n* = 1,000) were conducted for the means and error bars of feedback strength values. The column in white is for the same mycorrhizal type, the gray is for other mycorrhizal types and the black column represents eucalypt plants. An asterisk indicated a statistically significant negative or positive feedback values if their 95% confidence intervals (CIs) do not include zero. AM, arbuscular mycorrhizal; EM, ectomycorrhizal; NF, nitrogen-fixing; NM, non-mycorrhizal; EU, eucalypt; All, overall soil biota effect size across all heterospecific soil.

The PSF strength in heterospecific soil inocula depended on mycorrhizal type of both focal plants and soil origins (Figure 2). NM plants had consistent conspecific negative PSFs (i.e., indicating species coexistence); by contrast, NF and EU plants had consistently conspecific positive PSFs indicating they would not coexist with heterospecific plants. PSFs of AM and EM plants were variable with soil inocula from different mycorrhizal types. EU plants had consistent conspecific positive PSFs and induced negative PSFs for mycorrhizal plants (Figure 2). Different soil inocula did not result in significant difference on seedling survival (Supplementary Figure 4). There was a significant interaction between mycorrhizal type of focal plants and type of soil inocula on gainDW (Supplementary Table 3; *F* = 1.598, *P* = 0.042).

### PSF Experiment 2: Effects of Different Soil Microbial Compositions of *Eucalyptus*

**FIGURE 3:**
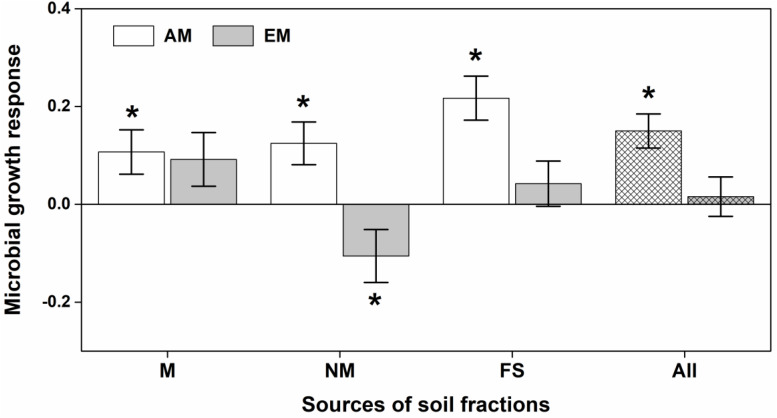
Effects of different microbial compositions in soil fractions on ratio of aboveground vs. underground biomass of arbuscular mycorrhizal (AM) and ectomycorrhizal (EM) plants. The microbial growth response (MGR) of seedlings was calculated as the log-response ratio comparing seedling growth between live soil fractions and sterilized soil fractions. M, NM, and FS were for mycorrhizal, non-mycorrhizal and field soil fractions respectively. All refers to overall soil biota effect size. MGR values above zero indicate positive effects of soil community on seedling growth, vice versa. An asterisk indicates a statistically significant negative or positive MGR value if their 95% confidence intervals (CIs) do not include zero.

Soil microbial community of *Eucalyptus* significantly altered biomass allocation (i.e., AbUn) of both AM and EM plants (Figure 3), but not for seedling biomass and seedling height (Supplementary Figure 7). AM seedlings consistently had positive MGRs across different microbial compositions of *Eucalyptus* soil biota, increasing more to the aboveground biomass; whereas, EM seedlings had a negative MGR when inoculated with non-mycorrhizal soil fractions of *Eucalyptus* (Figure 3).

## Discussion

Our observational and experimental results suggested that mycorrhizal-mediated PSF is able to drive species coexistence of tree species in *Eucalyptus* plantations. Our findings highlight the significant impact of compatibility between different mycorrhizal types on the survival and growth of tree species in mixed-species forest plantations. The strength and direction of PSFs depend on mycorrhizal types of focal species and soil inocula origins. *Eucalyptus* plants can induce unfavorable soil community resulting in conspecific positive PSFs on NF plants. NM plant would be a good candidate for established the multiple-species *Eucalyptus* plantations. Our findings reveal that compatibility between different mycorrhizal type plants may depend on the local adaptation to the specific soil microbial community cultured by distinct mycorrhizal plants.

Plants locally adapted to specific soil nutrient availability and chemistry and soil microbial community conditioned by plants with similar functional and phylogenetic traits (Johnson et al., 2010). Empirical evidence in natural plant communities has demonstrated the specialization of the soil microbial community in certain plant populations (Eck et al., 2019). The enemy release hypothesis is a potential explanation for the success of exotic plants in new habitats because of escape from their specific antagonist or natural enemies (Keane and Crawley, 2002). Exotic invasive plants can accumulate pathogens (Mangla and Callaway, 2008) or decrease mycorrhizal fungi (Stinson et al., 2006), and provide them with a competitive advantage over native species. Some researchers have shown exotic *Eucalyptus* can also alter the composition of soil microbial communities at the early developmental stage in subtropical China (Wu et al., 2013), resulting in changes in the local adaptation of native species to the soil community (Lankau, 2013). In this study, we found that *E. urophylla* cultured a very distinct soil microbial community with more unique microorganism species than other native species (Supplementary Figure 6). *Eucalyptus* seedlings were enhanced with its conspecific soil and had consistent conspecific positive feedbacks; however, there was no significant difference with the sterilized soil (Supplementary Figure 5). Together, *Eucalyptus*, as an exotic species, may not benefit from enemy release in our experimental region.

It is noted that *Eucalyptus* has been identified as invasive in some introduced regions, such as South Africa and the southeastern United States. However, *Eucalyptus* is not invasive in China since it was introduced in 1890s. According to our observation, it is not uncommon to find fallen dead trees of *E. urophylla* in the public welfare forest where our experimental plots are located. A recent study showed that PSF is a significant predictor of the success and even invasiveness of alien plants (Aldorfová et al., 2020). Our study also suggested the maladaptation to soil biota community of native species, rather than cumulation of pathogen, may account for the degradation of *Eucalyptus*. Consistently, the decreased local dominance of exotic plant species with time did not correlate with more negative PSF caused by soil pathogens (Speek et al., 2015).

Plant-plant interactions (e.g., facilitation and competition) play an important role in both facilitating and impeding species coexistence and the development of the forest community (Ibáñez and Rodríguez, 2020),which may depend on resource availability (Forrester et al., 2011). The functional compatibility or complementarity of mycorrhizal types plants, through culturing specific functional groups of soil microbes, contribute to relieve interspecific competition and promote species coexistence (Teste et al., 2014).

Mycorrhizal fungi, as an effective acquisition strategy for soil P, undergo significant community changes following conversion from secondary natural forest to exotic *Eucalyptus* plantations (Chen et al., 2018). The mycorrhizal fungal species abundance was significantly lower than that in the secondary forest soil and decreased with time after planting, and *Eucalyptus* plants had the lowest mycorrhizal inoculation rate over a relatively long-term period (∼10 a) (Li et al., 2019). Previous study has shown that adaptation among plants and locally adapted mycorrhiza’s and other root-associated fungi and rhizobacteria can help to predict the direction and outcome of PSFs (Revillini et al., 2016). The host-specific growth of mycorrhizal fungi reduces plant benefits and leads to negative feedback, decreasing plant performance within a mutualistic relationship (Bever, 2002). Hence, this degradation of exotic *Eucalyptus* plants may result from the incompatibility of mycorrhizal type for maladaptation to the local soil mycorrhizal community (Levine et al., 2004), because of less positive effects on plant growth mediated by P nutrient acquisition.

Interestingly, the strength and direction of PSFs may change during succession (Jing et al., 2015; Liao et al., 2018). The early-successional plants are generally fast growing and poorly defend against natural enemies, tend to have negative PSFs by its species-specific pathogens. Contrastingly, late-successional plants tend to more depend on mycorrhizal associations and have neutral to positive PSFs (Kardol et al., 2006). Alternatively, negative feedbacks may allow for early-successional species rather than late-successional species to coexist in early-successional plant communities as a result of the suppression of dominant species. On the contrary, our findings suggested that the late-successional non-mycorrhizal species (i.e., *H. cochinchinensis*) had consistent negative conspecific PSFs [i.e., log (Con/Heter)] and the highest survival rates in *Eucalyptus* plantations since planting, whereas the early-successional NF plants had consistent positive conspecific PSFs and the lowest survival rates (Figures 1, 2). The importance of different mycorrhizal types for increasing plant diversity is conventionally linked to the complementary use of soil resources such as soil N and P (Luo et al., 2018). Mixed plantations of *Eucalyptus* and NF trees is considered to enhance stand productivity by increase soil carbon and nitrogen pool (Santos et al., 2017; Pereira et al., 2019). We found that there was no significant difference on AN content in the species-specific soil of nine focal species. However, the content of P was relatively low in *Eucalyptus* soil (Supplementary Table 4). Soil P is the key limiting nutrient for plant growth in both tropical and subtropical forests (Condit et al., 2013). It also has a substantial impact on limiting *Eucalyptus* seedling growth in natural forest soil (Tng et al., 2014). Mycorrhizal association is an important strategies to enhance P acquisition by plants (Smith and Read, 2008). The variable pattern of AM and EM plants when inoculated with the soil community from different mycorrhizal types may increase plant diversity. Our previous study demonstrated that AM symbiosis can promote positive effects for native tree growth by counteracting the allelopathic inhibition of *Eucalyptus* (Qin and Yu, 2019). Interestingly, non-mycorrhizal species survived best in the *Eucalyptus* plantation (Figure 1). It has been demonstrated that non-mycorrhizal plants, such as *Proteaceae*, which may release large amounts of nutrient-mobilizing exudates from their cluster roots, tend to be common in a P-impoverished habitat (Lambers et al., 2006). We speculated that non-mycorrhizal plants had more efficient P acquisition due to their specialized cluster roots partitioning of soil P with other mycorrhizal plants.

Plant functional traits are important predictors for PSFs and able to shape the plant community and ecosystem functioning (Zirbel et al., 2017). The type of mycorrhizal association has been recently shown to determine the strength and direction of PSFs in several natural systems (Bennett et al., 2017; Zirbel et al., 2017),whereas the actual mechanism is still unidentified. Our study emphasized the possible mycorrhizal-mediated PSFs on species coexistence in mixed-species *Eucalyptus* plantations. However, limited to the time of pot experiments, only PSFs of seedlings at the early stage of growth were detected in pot experiments. And the pattern of seedling growth is vital for community succession and development. Species-specific traits of native tree species were conducive to change the competitive and facilitative effects of *Eucalyptus* on their survival and growth (Sun et al., 2017). Although the seedling survival and biomass growth are two important comprehensive indicators for performance, more physiological and nutrient parameters should be helpful to illustrate the interspecific interactions. Our findings in this study imply that *Eucalyptus* is more likely to be a selection pressure on soil microbes in a species-specific manner and drive PSFs among different mycorrhizal types. Therefore, more sophisticated experiments with method of radioactive element tracer, microbiome and metabonomics and more tree species are needed in the future. It will provide more mechanistical sight into PSFs among different mycorrhizal type trees.

## Conclusion

Our study provides consistent observational and experimental evidence for the importance of mycorrhizal type in the survival and growth of woody species in an afforestation system. Trees with distinct mycorrhizal associations give rise to soil microbial communities that differ in PSFs, which mediate their coexistence or exclusion. The results suggest that the strength and direction of PSFs of focal target tree species depend on mycorrhizal type. We illustrate how the compatibility among mycorrhizal types drive woody species coexistence through PSFs in mixed *Eucalyptus* plantations with native tree species. This is conductive to improve the silvicultural management of mixed-species forest plantations. Future studies will need to include more species for each mycorrhizal type and more systems may provide mechanistic insights into the species coexistence of woody species, which is important for making informed decisions on the selection of intercropped tree species for a better design for mixed-species forest plantations.

## Data Availability Statement

The original contributions presented in the study are included in the article/Supplementary Material, further inquiries can be directed to the corresponding author/s.

## Author Contributions

FQ wrote the first draft of the manuscript and conceived the experiment with advices from SY, conducted the experiment, and led data collection and analysis. FQ and SY contributed substantially to revisions. Both authors contributed to the article and approved the submitted version.

## Conflict of Interest

The authors declare that the research was conducted in the absence of any commercial or financial relationships that could be construed as a potential conflict of interest.

## Footnotes

1 http://ccb.jhu.edu/software/FLASH/

2 http://qiime.org/index.html

3 http://drive5.com/uparse/

4 http://sourceforge.net/projects/rdp-classifier/

5 http:/greengenes.lbl.gov/Download/
