# Supplementary material for: Compatible Mycorrhizal Types Contribute to a Better Design for Mixed Eucalyptus Plantations
Source: Front Plant Sci. 2021 Feb 12;12:616726. doi: 10.3389/fpls.2021.616726 (PMC7907608; doi:10.3389/fpls.2021.616726)
Supplement: Supplementary file 1 [file Data_Sheet_1.docx]

***Supplementary Material***

Table S1 List of the target plant species involved in the planting experiment (Field) and the two plant-soil feedback (PSF) experiments. These target species are belonged to different mycorrhizal types: (a) arbuscular mycorrhizal (AM), (b) ectomycorrhizal (EM), (c) nitrogen-fixing (NF), (d) nonmycorrhizal (NM) and (e) eucalyptus (EU).

| No | Codes | Species | Family | Mycorrhizal type | Succession place | Field | PSF | |
| --- | --- | --- | --- | --- | --- | --- | --- | --- |
|  |  |  |  |  |  |  | 1 | 2 |
| 1 | AcAc | *Acmena acuminatissima* | Myrtaceae | AM | Late | 🗸 |  |  |
| 2 | AlLe | *Albizia lebbeck* | Leguminosae | NF | Early | 🗸 | 🗸 |  |
| 3 | CaAl | *Canarium album* | [Burseraceae](http://www.iplant.cn/info/Burseraceae?t=z) | AM | Late |  |  | 🗸 |
| 4 | CaCh | *Castanopsis chinensis* | Fagaceae | EM | Early | 🗸 | 🗸 | 🗸 |
| 5 | CaFa | *Castanopsis faberi* | Fagaceae | EM | Early |  |  | 🗸 |
| 6 | CaFi | *Castanopsis fissa* | Fagaceae | EM | Early | 🗸 | 🗸 | 🗸 |
| 7 | CiBu | *Cinnamomum burmanni* | Lauraceae | AM | Late | 🗸 |  |  |
| 8 | CiCa | *Cinnamomum camphora* | Lauraceae | AM | Early | 🗸 | 🗸 | 🗸 |
| 9 | CrCo | *Cryptocarya concinna* | Lauraceae | AM | Late | 🗸 |  |  |
| 10 | CyBa | *Cyclobalanopsis bambusaefolia* | [Fagaceae](http://www.iplant.cn/info/Fagaceae?t=z) | EM | Late |  |  | 🗸 |
| 11 | CyFl | *Cyclobalanopsis fleuryi* | [Fagaceae](http://www.iplant.cn/info/Fagaceae?t=z) | EM | Early |  |  | 🗸 |
| 12 | CyHu | *Cyclobalanopsis hui* | Fagaceae | EM | Late | 🗸 |  |  |
| 13 | CyMy | *Cyclobalanopsis myrsinaefolia* | Fagaceae | EM | Late | 🗸 |  |  |
| 14 | EuUr | *Eucalyptus urphylla* | Myrtaceae | EU | Early |  | 🗸 |  |
| 15 | HeCo | *Helicia cochinchinensis* | Proteaceae | NM | Late | 🗸 | 🗸 |  |
| 16 | HoDu | *Hovenia dulcis* | Rhamnaceae | AM | Late | 🗸 |  | 🗸 |
| 17 | IiTr | *Ilex triflora* | Aquifoliaceae | AM | Late | 🗸 |  |  |
| 18 | LeLe | *Leucaena leucocephala* | Leguminosae | NF | Early | 🗸 |  |  |
| 19 | MaCh | *Machilus chinensis* | Lauraceae | AM | Late | 🗸 |  |  |
| 20 | MiMa | *Michelia maclurei* | Magnoliaceae | AM | Early | 🗸 |  |  |
| 21 | OrGl | *Ormosia glaberrima* | Leguminosae | NF | Late | 🗸 | 🗸 |  |
| 22 | PhBe | *Photinia benthamiana* | Rosaceae | AM | Late | 🗸 |  |  |
| 23 | PtLa | *Pterospermum lanceaefolium* | Sterculiaceae | AM | Late | 🗸 | 🗸 |  |
| 24 | ScOc | *Schefflera octophylla* | Araliaceae | AM | Late | 🗸 |  |  |
| 25 | ScSu | *Schima superba* | Theaceae | AM | Early | 🗸 | 🗸 |  |

*Note:* (1) Although some species are dual-mycorrhizal plants, we focused only on the most abundant mutualist type encountered. For example, the leguminous plants were categorized as NF species rather than AM species. (2) *Eucalyptus* plants are able to simultaneously colonize with both AM fungi and EM fungi; and what we focused in this study is the mechanism underpinning species coexistence in the context of *Eucalyptus* plantations. Thus, we listed mycorrhizal association type of *Eucalyptus* plants as a category of “EU”. (3) *H. cochinchinensis* has specialized cluster roots and we categorized it as NM plant. (4) We specified those focal species as early-successional species or late-successional species, according to results in “Flora Reipublicae Popularis Sinicae” (http://www.cn-flora.ac.cn/) and our field observations.


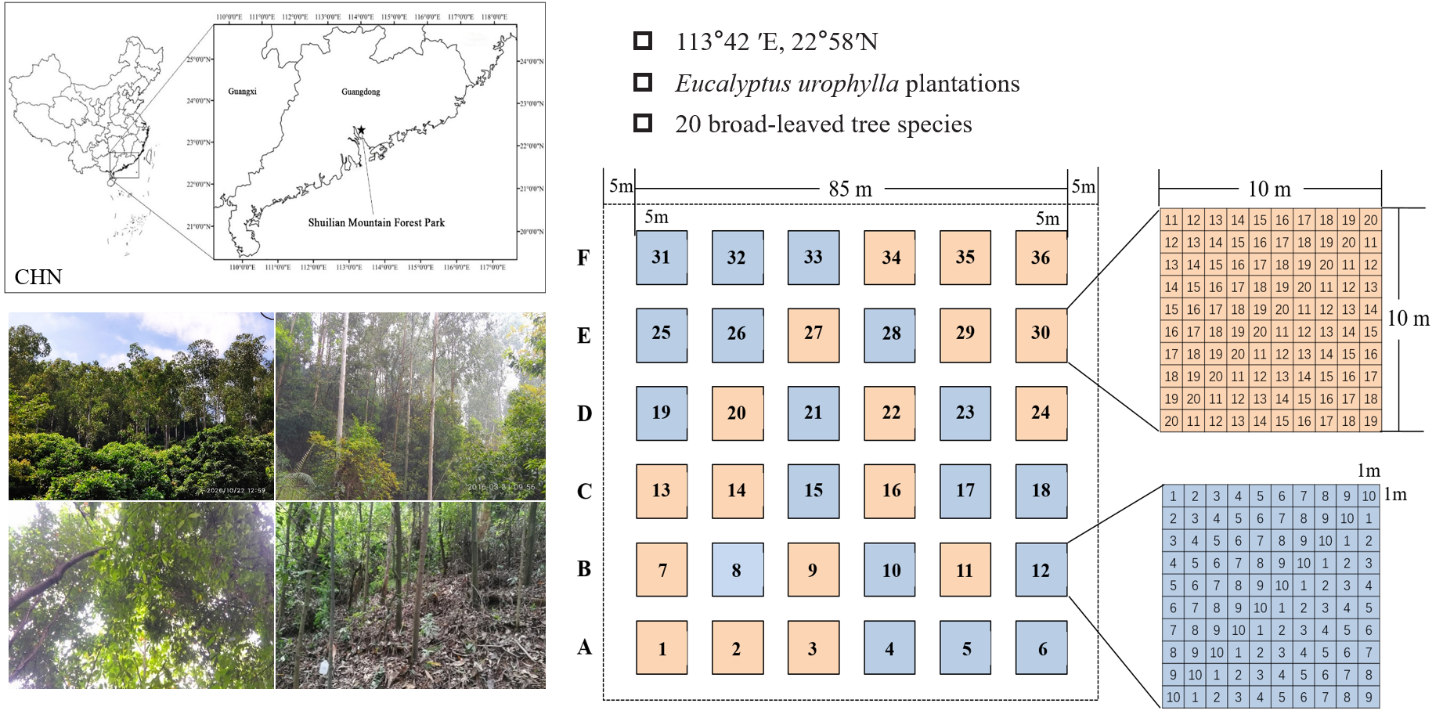


Figure. S1 Details of permanent plot and design for planting experiment. We established 6 transects (15 m × 95 m) in the *E. urophylla* plantations during winter 2006 to spring 2007, along the contours of the hillside. Each transect included 6 plots (10 m × 10 m) with 5 m-wide buffer zones between each plot. We cleared all understory vegetations and left 4-6 *E. urophylla* trees. In every transect, we separately transplanted seedlings of 10 tree species into every 3 out of 6 plots, at a density of 1 seedling/m^2^ (i.e., 100 seedlings per plot); the other 10 species were transplanted into the rest of 3 plots as the same method.


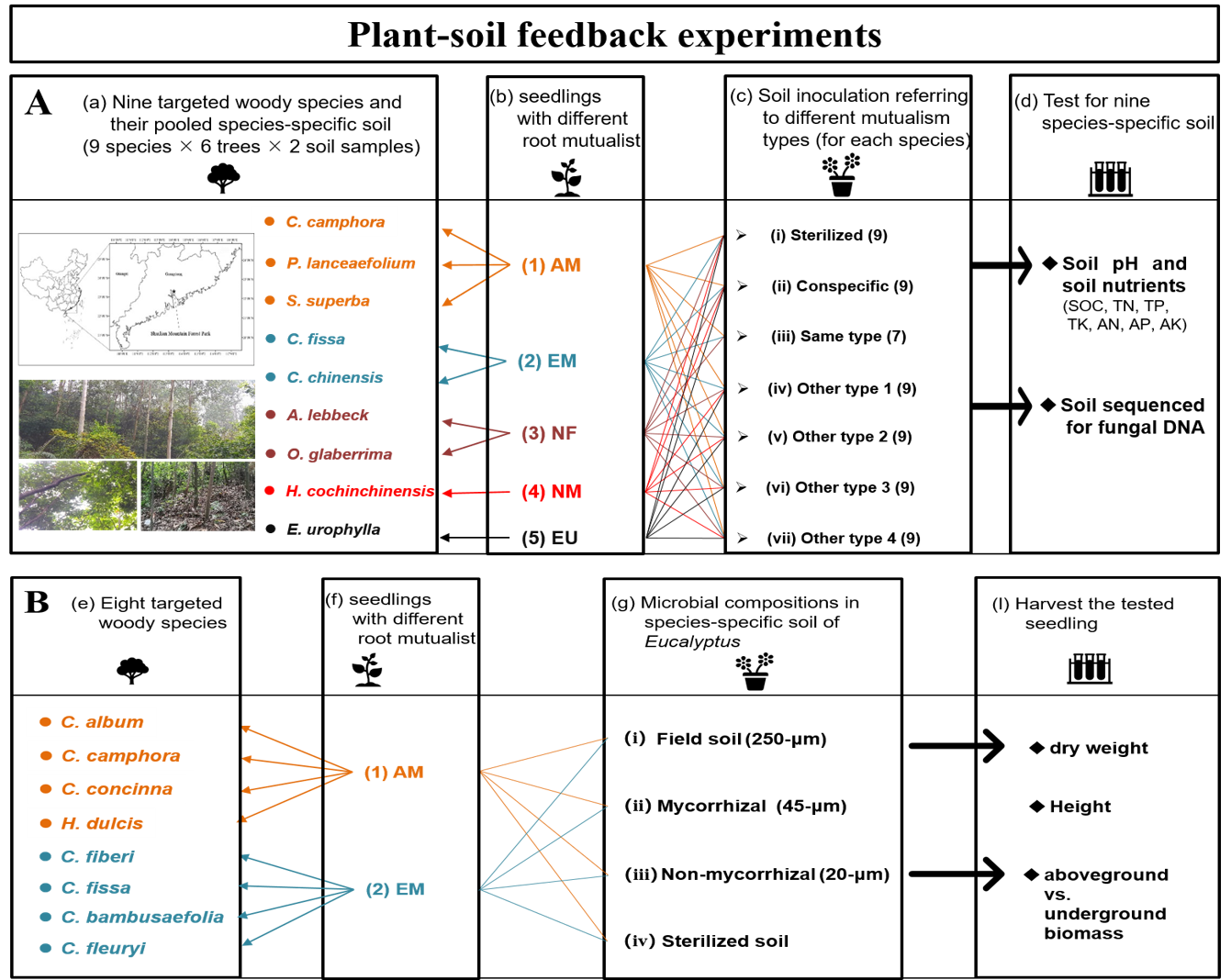


Figure. S2 Schematic design for the PSF *Experiment 1* (A) and *Experiment 2* (B). (A) We prepared 9 species-specific soil inocula by pooling soil from 6 adult individuals for each focal species, which referred to 5 distinct mycorrhizal types, namely arbuscular mycorrhizal (AM, orange), ectomycorrhizal (EM, blue), nitrogen-fixing (NF, purple), non-mycorrhizal (NM, red) and eucalyptus (EU, black). We determined the [physicochemical](javascript:;) [property](javascript:;) and characterized their soil fungal communities using next-generation sequencing. And then, we separately pooled the species-specific soil according to different mycorrhizal types and lead to 6 or 7 soil inocula for targeted species as follows: (i) sterilized, (ii) conspecific, (ii) same type with focal species and (iv)-(vii) the other 4 contrasting mycorrhizal types. (B) To test effects of different microbial compositions in *Eucalyptus* soil, we chose four AM (i.e.， *C. album*, *C. camphora*, *C. concinna*, *H. dulcis*) and four EM (i.e.， *C. faberi*, *C. fissa*, *C.* *bambusaefolia*, *C. fleuryi*) woody species as focal plants. We separated the microbial composition in soil wet-sieving method. Focal plants were inoculated with the sterilized soil, soil with mycorrhizal fungi (45-μm), soil without mycorrhizal fungi (20-μm) and field soil (250-μm) fractions from adult tree of *Eucalyptus*. SOC, soil organic carbon; TN, total nitrogen; TP, total phosphorus; TK, total potassium; AN, available nitrogen; AP, available phosphorus; AK, available potassium.





Figure. S3 Survival rates of 20 target species at different time after transplanting into the *Eucalyptus* plantations. The 20 tested species as follow: *P. benthamiana*, PhBe; *C. chinensis*, CaCh; *S. octophylla*, ScOc; *A. acuminatissima*, AcAc; *C. fissa*, CaFi; *I. triflora*, IiTr; *P. lanceaefolium*, PtLa; *C. concinna*, CrCo; *L. leucocephala*, LeLe; *A. lebbeck*, AlLe; *C. camphora*, CiCa; *S. superba*, ScSu; *C. hui*, CyHu; *C. burmanni*, CiBu; *H. cochinchinensis*, HeCo; *C. myrsinaefolia*, CyMy; *M. chinensis*, MaCh; *H. dulcis*, HoDu; *O. glaberrima*, OrGl; *M. maclurei*, MiMa.

Table S2 ANOVA test assessing the effects of mycorrhizal type on seedling survival and basal diameter growth in the field.

| Index | 18 months | |  | 36 months | |  | 54 months | |  | 114 months | |
| --- | --- | --- | --- | --- | --- | --- | --- | --- | --- | --- | --- |
|  | F | *P* |  | F | *P* |  | F | *P* |  | F | *P* |
| Seedling survival | 104.28 | < 0.001 |  | 103.33.00 | < 0.001 |  | 111.24 | < 0.001 |  | 73.677 | < 0.001 |
| Growth of basal diameter | 15.47 | < 0.001 |  | 33.85 | < 0.001 |  | 67.93 | < 0.001 |  | 67.85 | < 0.001 |

Table S3 [Variance](javascript:;) [analysis](javascript:;) results of fixed effects of mycorrhizal type (M), inocula source (I) and their interactions (M × I) on the gain of dry weight (gainDW), height (gainH), number of leaf (gainNL), specific leaf area (SLA) and ratio of above-/under-ground biomass (AbUn) of target species in the PSF *Experiment 1*.

| Index | Mycorrhizal type (M) | |  | Inocula source (I) | | M × I | |
| --- | --- | --- | --- | --- | --- | --- | --- |
|  | F | *P* |  | F | *P* | F | *P* |
| gainDW | 47.559 | 0.000 |  | 3.421 | 0.003 | 1.598 | 0.042 |
| gainH | 56.252 | 0.000 |  | 1.504 | 0.175 | 1.203 | 0.240 |
| gainNL | 143.703 | 0.000 |  | 2.677 | 0.015 | 1.118 | 0.323 |
| SLA | 70.679 | 0.000 |  | 0.553 | 0.768 | 0.708 | 0.828 |
| AbUn | 79.645 | 0.000 |  | 5.022 | 0.000 | 0.786 | 0.745 |





Figure. S4 Seedling survival in relation to mycorrhizal type (in PSF *Experiment 1*). Con, conspecific; AM, arbuscular mycorrhizal; EM, ectomycorrhizal; NF, nitrogen-fixing; NM, non-mycorrhizal; EU, eucalyptus; Strl, sterile conspecific.





Figure. S5 Dry weight gain (a), height gain (b) and specific leaf area (c) of focal plants inoculated with soil from different mycorrhizal types or sterilized soil (in PSF *Experiment 1*). The values were represented as mean ± SE for per plant individual. Different letters indicate statistically significant differences (*P* < 0.05). Con, conspecific; AM, arbuscular mycorrhizal; EM, ectomycorrhizal; NF, nitrogen-fixing; NM, non-mycorrhizal; EU, eucalyptus; Strl, sterile conspecific.

Table S4 Soil inocula characteristics of 9 target species including *C. camphora* (CiCa), *P. lanceaefolium* (PtLa), *S. superba* (ScSu), *C. fissa* (CaFi), *C. chinensis* (CaCh), *A. lebbeck* (AlLe), *O. glaberrima* (OrGl), *H. cochinchinensis* (HeCo) and *E. urophylla* (EuUr).

|  | Codes | pH | SOC (g/kg) | TN (g/kg) | TP (g/kg) | TK (g/kg) | AN (mg/kg) | AP (mg/kg) | AK (mg/kg) |
| --- | --- | --- | --- | --- | --- | --- | --- | --- | --- |
|  |  |  |  |  |  |  |  |  |  |
| 1 | CiCa | 4.27 d | 33.90 bc | 1.63 b | 0.31 a | 14.52 bc | 151.90 a | 2.07 e | 77.42 c |
| 2 | PtLa | 4.43 a | 32.12 bc | 1.54 bc | 0.32 a | 16.62 a | 142.20 a | 2.70 de | 82.22 cd |
| 3 | ScSu | 4.35 b | 31.11 cd | 1.65 b | 0.30 a | 15.83 ab | 145.03 a | 4.10 bcd | 87.72 ab |
| 4 | CaFi | 4.23 e | 36.93 a | 2.03 a | 0.33 a | 14.71 bc | 165.03 a | 3.67 cd | 91.68 a |
| 5 | CaCh | 4.34 bc | 32.65 bc | 1.34 cd | 0.34 a | 15.24 bc | 147.03 a | 7.90 a | 88.79 ab |
| 6 | AlLe | 4.46 a | 28.49 d | 1.20 d | 0.32 a | 15.50 ab | 148.47 a | 5.30 b | 87.58 ab |
| 7 | OrGl | 4.38 b | 33.26 bc | 1.38 cd | 0.31 a | 15.32 bc | 144.47 a | 5.33 b | 84.60 bc |
| 8 | HeCo | 4.30 cd | 35.09 ab | 1.31 cd | 0.32 a | 14.15 c | 152.73 a | 4.90 bc | 78.81 c |
| 9 | EuUr | 4.17 f | 37.94 a | 1.36 cd | 0.26 b | 14.86 bc | 152.77 a | 1.77 e | 78.41 c |

*Note*: SOC, soil organic carbon; TN, total nitrogen; TP, total phosphorus; TK, total potassium; AN, available nitrogen; AP, available phosphorus; AK, available potassium.


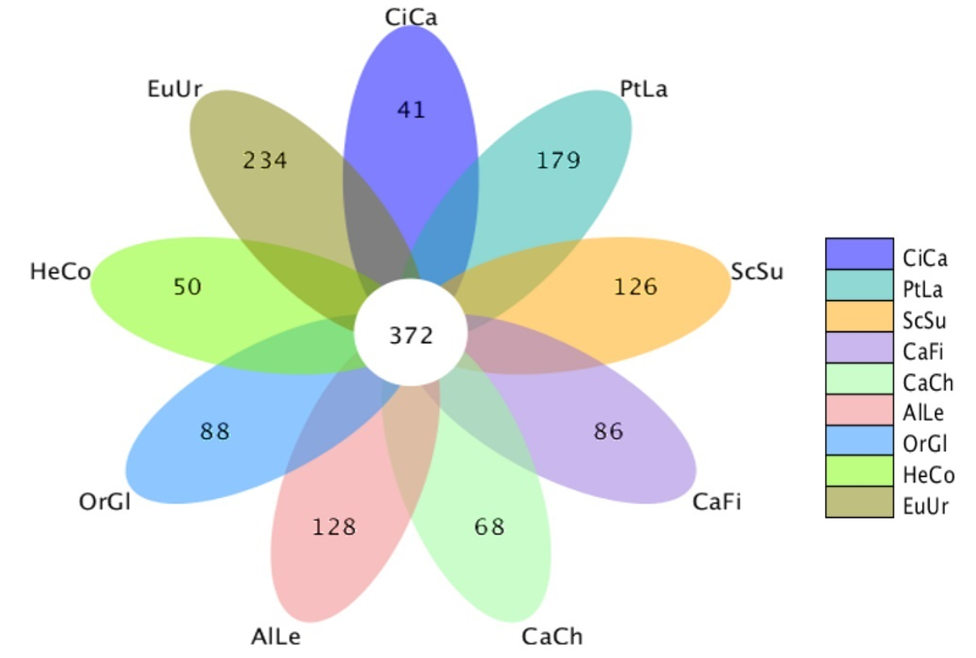


Figure. S6 The numerical relationship of Operational Taxonomic Units (OTUs, referring to fungal species) for species-specificity of 9 targeted species. The digits in figure represent the number of unique (in the outline) or shared OTUs (in the core), respectively. *C. camphora*, CiCa; *P. lanceaefolium*, PtLa; *S. superba*, ScSu; *C. fissa*, CaFi; *C. chinensis*, CaCh; *A. lebbeck*, AlLe; *O. glaberrima*, OrGl; *H. cochinchinensis*, HeCo; *E. urophylla*, EuUr.



 Figure. S7 Effects of different microbial compositions in soil inocula on seedling biomass (a) and seedling height (b) of arbuscular mycorrhizal (AM) and ectomycorrhizal (EM) plants (in PSF *Experiment 2*). The microbial growth response (MGR) of seedlings was calculated as the log-response ratio comparing seedling growth between live soil fractions and sterilized soil fractions. M, NM and FS were for mycorrhizal, non-mycorrhizal and field soil fractions respectively; All refers to overall soil biota effect size. MGR values above zero indicate positive effects of soil community on seedling growth, vice versa. An asterisk indicates a statistically significant negative or positive MGR value if their 95% confidence intervals (CIs) do not include zero.
